# Deeplasia: deep learning for bone age assessment validated on skeletal dysplasias

**DOI:** 10.1007/s00247-023-05789-1

**Published:** 2023-11-13

**Authors:** Sebastian Rassmann, Alexandra Keller, Kyra Skaf, Alexander Hustinx, Ruth Gausche, Miguel A. Ibarra-Arrelano, Tzung-Chien Hsieh, Yolande E. D. Madajieu, Markus M. Nöthen, Roland Pfäffle, Ulrike I. Attenberger, Mark Born, Klaus Mohnike, Peter M. Krawitz, Behnam Javanmardi

**Affiliations:** 1 Institute for Genomic Statistics and Bioinformatics, University Hospital Bonn, Venusberg-Campus 1 Building 11, 2nd Floor, 53127 Bonn, Germany; 2 Kinderzentrum Am Johannisplatz, Leipzig, Germany; 3 Medical Faculty, Otto-Von-Guericke-University Magdeburg, Magdeburg, Germany; 4 CrescNet - Wachstumsnetzwerk, Medical Faculty, University Hospital Leipzig, Leipzig, Germany; 5 Institute of Human Genetics, University Hospital Bonn, Bonn, Germany; 6 Department for Pediatrics, University Hospital Leipzig, Leipzig, Germany; 7 Department of Diagnostic and Interventional Radiology, University Hospital Bonn, Bonn, Germany; 8 Division of Paediatric Radiology, Department of Radiology, University Hospital Bonn, Bonn, Germany

**Keywords:** Artificial intelligence, Bone age measurement, Bone dysplasias, Children, Deep learning, Genetic diseases, Rare diseases

## Abstract

**Background:**

Skeletal dysplasias collectively affect a large number of patients worldwide. Most of these disorders cause growth anomalies. Hence, evaluating skeletal maturity via the determination of bone age (BA) is a useful tool. Moreover, consecutive BA measurements are crucial for monitoring the growth of patients with such disorders, especially for timing hormonal treatment or orthopedic interventions. However, manual BA assessment is time-consuming and suffers from high intra- and inter-rater variability. This is further exacerbated by genetic disorders causing severe skeletal malformations. While numerous approaches to automate BA assessment have been proposed, few are validated for BA assessment on children with skeletal dysplasias.

**Objective:**

We present Deeplasia, an open-source prior-free deep-learning approach designed for BA assessment specifically validated on patients with skeletal dysplasias.

**Materials and methods:**

We trained multiple convolutional neural network models under various conditions and selected three to build a precise model ensemble. We utilized the public BA dataset from the Radiological Society of North America (RSNA) consisting of training, validation, and test subsets containing 12,611, 1,425, and 200 hand and wrist radiographs, respectively. For testing the performance of our model ensemble on dysplastic hands, we retrospectively collected 568 radiographs from 189 patients with molecularly confirmed diagnoses of seven different genetic bone disorders including achondroplasia and hypochondroplasia. A subset of the dysplastic cohort (149 images) was used to estimate the test–retest precision of our model ensemble on longitudinal data.

**Results:**

The mean absolute difference of Deeplasia for the RSNA test set (based on the average of six different reference ratings) and dysplastic set (based on the average of two different reference ratings) were 3.87 and 5.84 months, respectively. The test–retest precision of Deeplasia on longitudinal data (2.74 months) is estimated to be similar to a human expert.

**Conclusion:**

We demonstrated that Deeplasia is competent in assessing the age and monitoring the development of both normal and dysplastic bones.

**Graphical Abstract:**

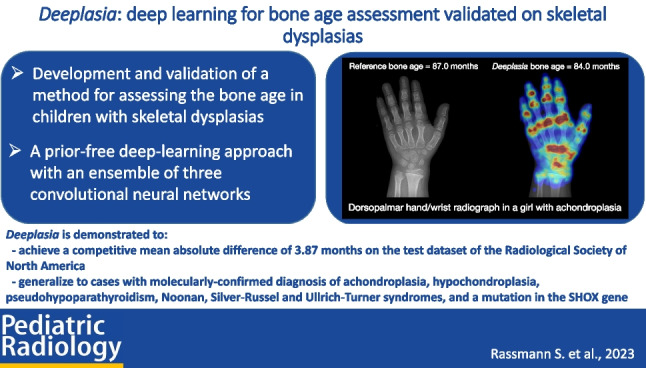

**Supplementary Information:**

Supplementary material is available at 10.1007/s00247-023-05789-1.

## Introduction

The estimation of bone age (BA), which evaluates skeletal maturity, is a valuable tool in assessing children’s growth. Usually, it is one of the first steps in the diagnosis of pediatric growth disorders [1]. In particular, for conditions in which hormonal therapy or orthopedic interventions are being considered, the timing of the treatment depends on the assessed BA [2]. The BA can be estimated by observing the ossification centers of a child’s skeleton. The main body parts used for BA assessment are the hands, wrists, and knees. BA estimates from the hand and wrist are more closely correlated with the child’s overall growth progress and puberty onset than estimates from the knee. Hence, the BA estimated from hand radiographs is more effective in assessing delayed or advanced growth [3] and is therefore used as a routine diagnostic and monitoring method [4, 5]. The Greulich-Pyle (GP) [6] and Tanner-Whitehouse (TW) [7–9] are the two most commonly used hand and wrist BA estimation methods. While the TW method is considered to be more accurate, the GP method is generally regarded to be faster [10]. Nevertheless, both methods are time-consuming and show high degrees of inter- and intra-rater variability [10, 11].

Artificial intelligence (AI) methods contribute to all medical fields [12] including pediatric radiology [13] and numerous machine learning (ML) approaches have been proposed to automate BA assessment, most of them relying on a publicly available dataset released in 2017 by the Radiological Society of North America (RSNA) for their pediatric BA challenge [14, 15]. While an approach using end-to-end deep learning (DL) without any prior input, e.g., specific regions of interest (ROIs) or a particularly task-specific design, won the competition [15, 16], ML approaches emphasizing anatomical features used in human BA assessment have shown some improvement in more recent studies [17–20].

A major indication to perform BA assessments is suspected growth or developmental anomalies. This is often connected to the phenotype of skeletal dysplasias [21], which are rare genetic disorders. Although these disorders are individually rare [22], collectively they affect a large number of children [23] with an estimated total number of around 25 million worldwide. Especially in such patients, reliable and precise BA estimations are important for the initial assessment and monitoring of the maturation progress over time [24]. As skeletal dysplasias alter hand morphology, conventional methods relying on the identification of individual bones or ROIs might be unsuitable for precise BA assessment. For example, the commonly-used BA assessment tool BoneXpert (Visiana, Hørsholm, Denmark, [25]) struggles to generalize to all patients with skeletal dysplasias and, for example, rejects around 50% of cases with achondroplasia (personal communication with H. H. Thodberg, March 2023). However, this problem is still understudied because many approaches to automatic BA assessment have been developed for and tested on datasets composed of predominantly normally-developing children. The public dataset released as part of the 2017 BA challenge contains only 0*.*21% cases of reported skeletal dysplasias [14, 15] and the more recent study by Thodberg et al. [25] included <1*.*4% of patients with congenital diseases. Kim et al. [26] and Wang et al. [27] proposed and tested DL methods on patients with abnormal growth; however, their study was limited to Korean and Chinese populations, respectively, and their test sets included no or only small numbers (*n*<10) of images from patients with severe skeletal dysplasias such as achondroplasia.

In this article, we introduce Deeplasia: an AI application designed for BA assessment specifically validated on the hands of patients with skeletal dysplasias. Given the intrinsic scarcity of data from patients with rare diseases, our aim was to present an open-source tool that, while trained on data of normal hands, can reliably be used for assessing BA of patients with rare bone diseases.

## Materials and methods

### Training and validation datasets

We used the 2017 RSNA training and validation sets containing 12,611 and 1,425 images, respectively. RSNA published these data for their Pediatric BA ML Challenge [14, 15]. The data were obtained from Children’s Hospital Colorado (Aurora, CO) and Lucile Packard Children’s Hospital at Stanford (Palo Alto, CA). For each image, the sex and a ground truth GP BA estimate are provided. For determining the ground truth BA, one estimate from the original clinic providing the data, a second estimate from the same rater at least 1 year later, and four independent estimates were obtained. To form the final consensus BA estimate, a weighted mean based on the performance of each reviewer is calculated (for more details, see Halabi et al. [15]). The mean chronological age of patients in the training and validation set is 10*.*8 ± 3*.*5 years [14] and their mean estimated BA is 10*.*6 ± 3*.*4 years [15].

### Test datasets

For validating our AI, we used three independent test datasets as described below.

#### The test set from the Radiological Society of North America

The RSNA test dataset from the Pediatric BA ML Challenge [14, 15] contains 200 images (100 males) from Lucile Packard Children’s Hospital. The mean chronological age of patients in this set is 11*.*3 ± 3*.*8 years [14] and the mean estimated BA is 11*.*0 ± 3*.*6 years [15]. Similar to the training and validation sets, the sex and a ground truth GP BA estimate (weighted average of six measurements) are provided for each image. The distribution of ground truth BA for males and females in the test set is similar to those in the training and validation sets.

#### Los Angeles Digital Hand Atlas

As an additional test set for normally-developing children, we used the publicly released Los Angeles Digital Hand Atlas [28, 29]. It consists of 1,390 images acquired between 1997 and 2008 at the Children’s Hospital Los Angeles, United States of America. The study cohort included four ethnicities and ground truth BA estimates were obtained by two raters using the GP atlas. The ground truth BA was defined as the average of the two ratings. We excluded seven images due to lacking or completely implausible ground truth BA assessment (BA of 99 years, BA of 0 years for children with chronological age of 9 years, and two images with a difference compared to a third manual assessment by K.M., [a pediatric endocrinologist with more than 40 years of clinical experience] of >2 years).

#### German Dysplastic Bone Dataset

To compile a dataset for validating the BA prediction models on dysplastic hands, we retrospectively collected hand radiographs from patients referred to the pediatric endocrinology of two German university hospitals (Magdeburg and Leipzig) due to a suspected growth disorder between 2006 and 2022. The radiographs were acquired as hard copies and thereafter digitized. The study was approved by the ethics committee of the medical faculties of the universities of Magdeburg (reference 27/22) and Leipzig (reference 121/22-ek).

**Fig. 1:**
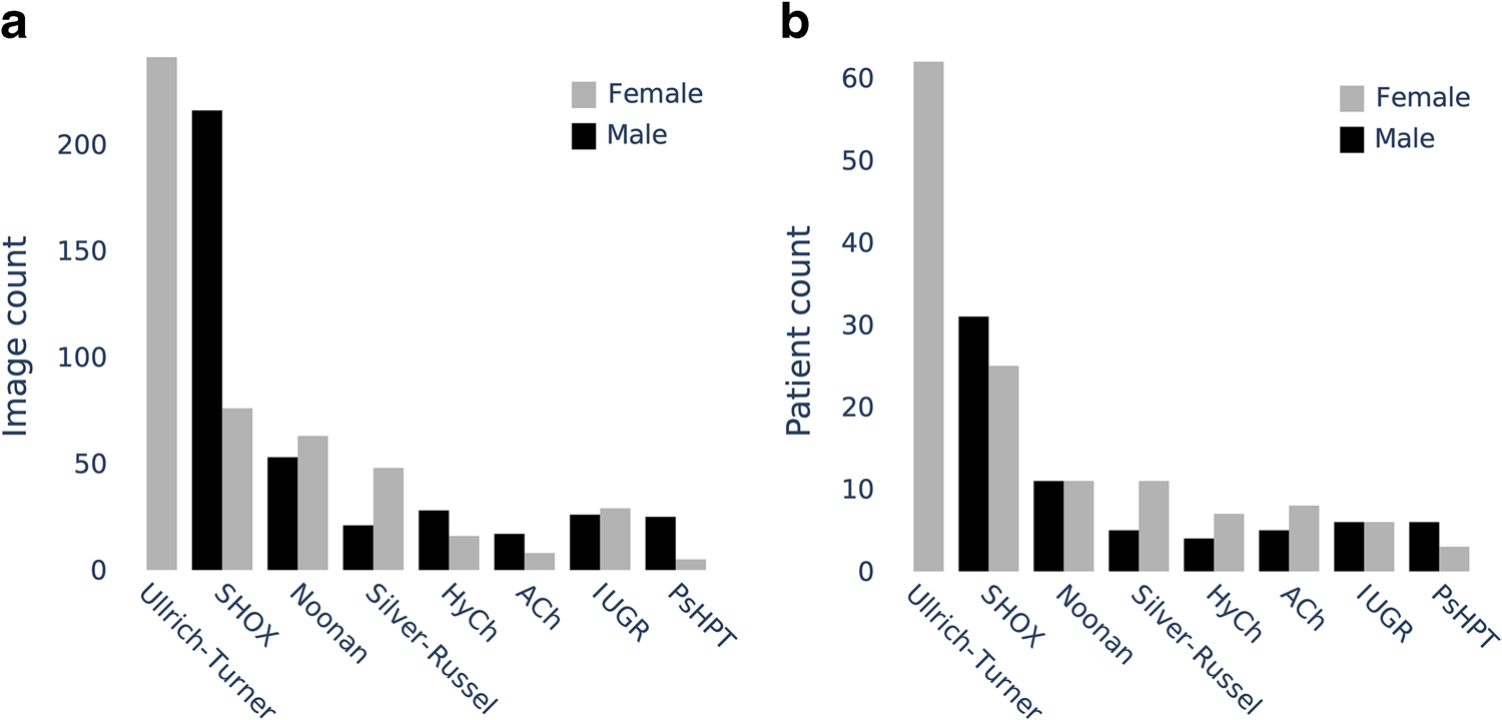
The German Dysplastic Bone Dataset distribution of image (**a**) and patient (**b**) counts for specific disorders. *ACh* achondroplasia, *HyCh* hypochondroplasia, *IUGR* intrauterine growth restriction, *PsHPT* pseudohypoparathyroidism

**Fig. 2:**
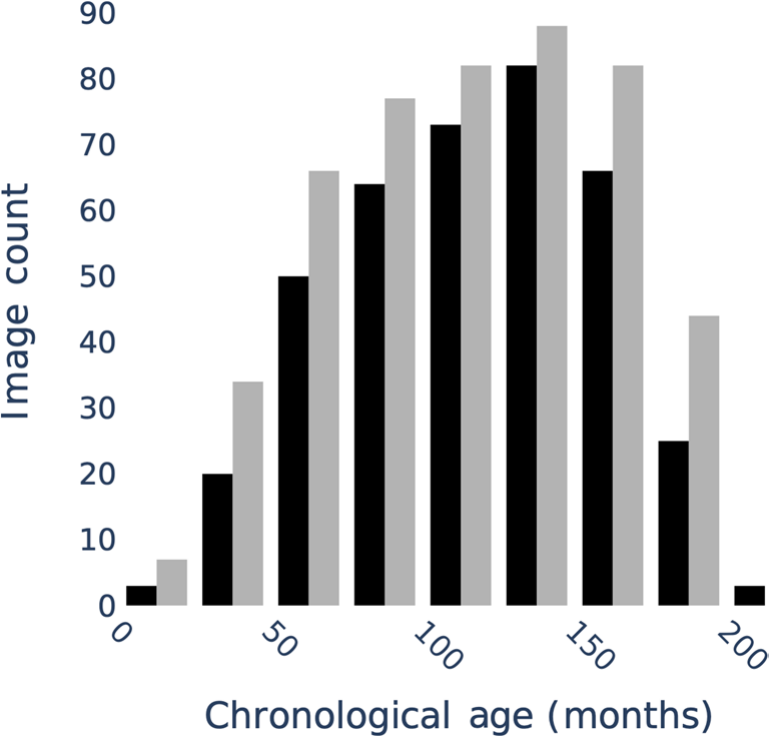
The cross-cohort distribution of chronological age in the German Dysplastic Bone Dataset

**Fig. 3:**
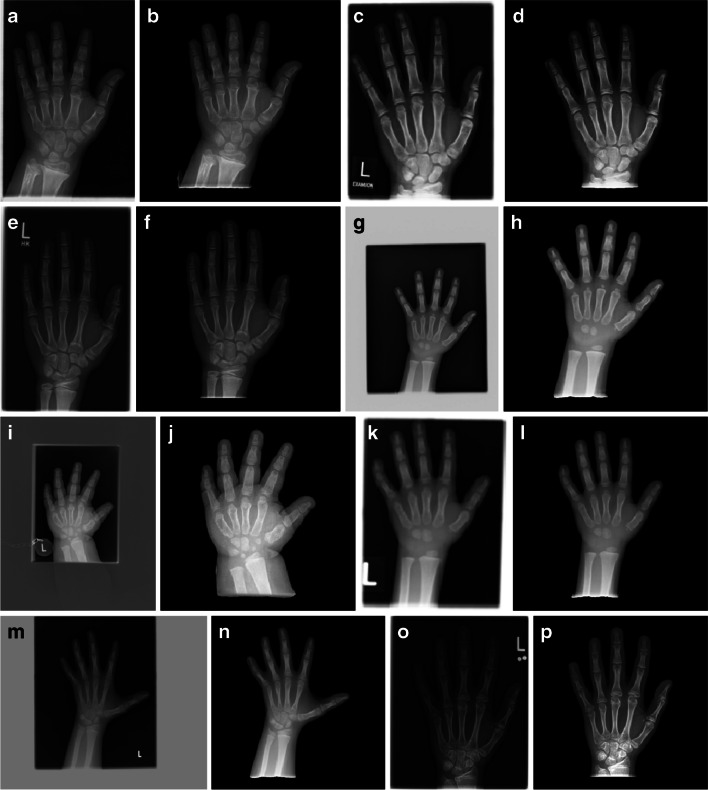
Example dorsopalmar hand and wrist radiographs for each bone dysplasia in the German Dysplasia Bone Dataset. **a**, **b** A 10-year-old boy with achondroplasia. **c**, **d** A 10-year-old girl with hypochondroplasia. **e**, **f** A 12-year-old boy with intrauterine growth restriction. **g**, **h** A 3-year-old boy with Noonan syndrome. **i**, **j** A 4-year-old girl with pseudohypoparathyroidism. **k**, **l** A 4-year-old boy with a mutation of the *SHOX* gene. **m**, **n** A 9-year-old girl with Silver-Russell syndrome. **o**, **p** A 14-year-old girl with Ullrich-Turner syndrome. For each example, the original raw (**a**, **c**, **e**, **g**, **i**, **k**, **m**, **o**) and preprocessed (**b**, **d**, **f**, **h**, **j**, **l**, **n**, **p**) image is shown. There is a wide range of relative scales within the images; image qualities vary and show artifacts such as labels and white or gray backgrounds caused by scanning

We term this dataset the German Dysplastic Bone Dataset (GDBD). In total, it contains 568 hand radiographs from 189 patients with molecularly confirmed diagnoses of one of the following disorders: achondroplasia; hypochondroplasia; pseudohypoparathyroidism; Noonan, Silver-Russell, and Ullrich-Turner syndromes; and a mutation in the *SHOX* gene. Further, to increase the diversity of this dataset, we supplemented it with 55 images from 12 patients with intrauterine growth restriction (IUGR) and 79 images from 79 children who were suspected to have a growth anomaly but had not been genetically diagnosed with any skeletal dysplasia. The number of images and patients and the distribution of their chronological age are shown in Figs. 1 and 2, respectively. An example hand and wrist radiograph of each disorder is shown in Fig. 3. The ethnic background of these patients is not available; however, we suspect a large portion of them to be Caucasian.

The BA reference gradings for the German Dysplastic Bone Dataset were obtained using the GP standard by K.M. and A.K. (a pediatric endocrinologist with more than 20 years of clinical experience). For 643 out of 702 images, one of the assessments was obtained from the initial clinical report (by a pediatric radiologist or endocrinologist). The BA ratings for the remaining images and the second reference ratings were obtained from a dedicated session, in which the images were presented (a) using the same preprocessing procedures as for testing the models, (b) in a randomized order, and (c) blinded for the chronological age, the clinical report, and the diagnosis.

**Fig. 4:**
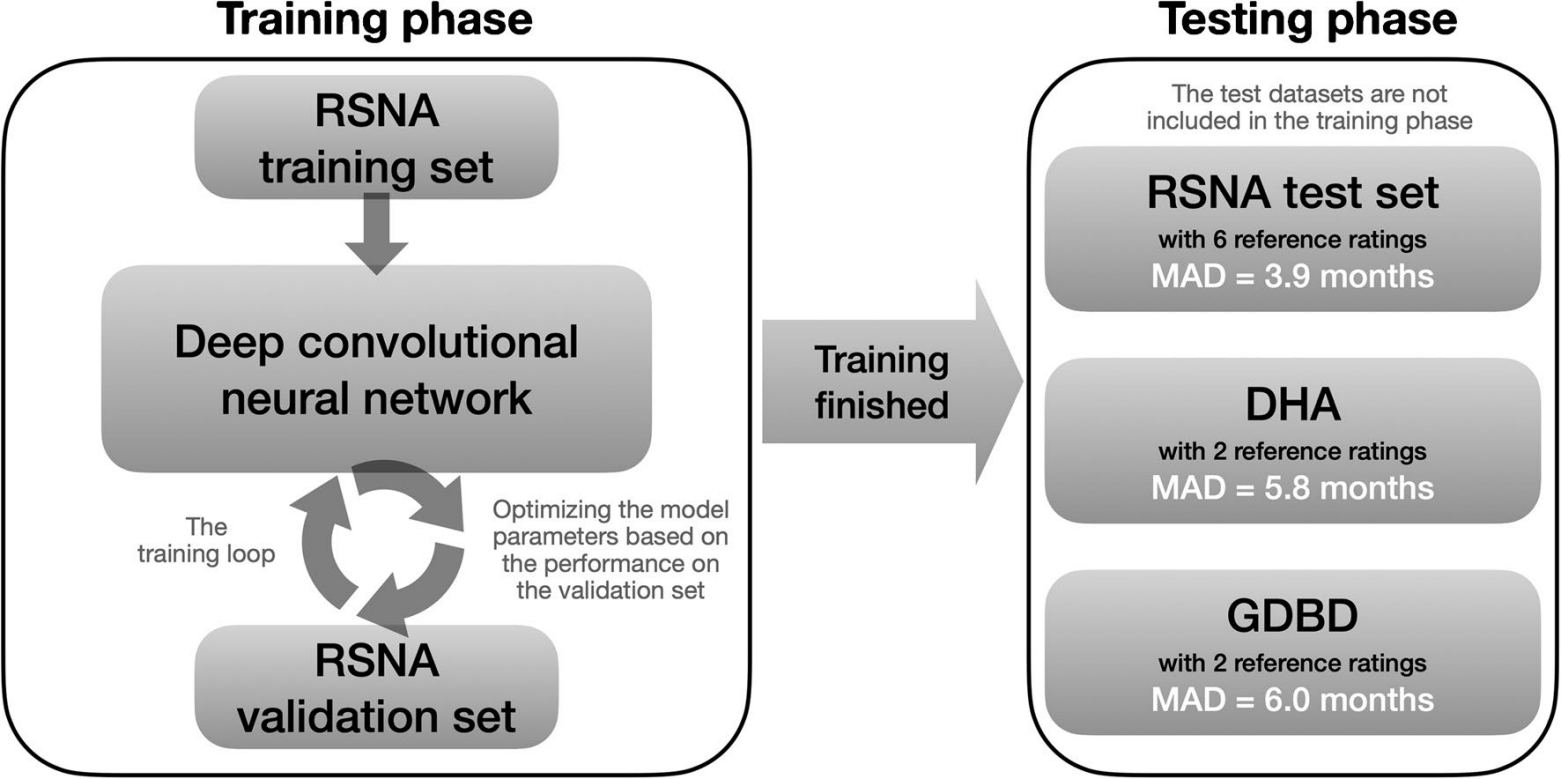
Flowchart describing the use of the different datasets for training and testing of our models. *DHA* Los Angeles Digital Hand Atlas, *GDBD* German Dysplastic Bone Dataset, *MAD* mean absolute difference, *RSNA* Radiological Society of North America

The process by which the datasets described above were used in the training and testing of our AI is shown in Fig. 4.

### Design and development of Deeplasia

#### Image background removal and preprocessing

Imaging and scanning induce artifacts to the input images, e.g., department-specific markers or white borders surrounding the radiograph carrier in the scan. Such artifacts (which are present in many of the images in our German Dysplastic Bone Dataset) have been shown to bias DL models, for example by Zech et al. [30]. Further, high-intensity borders can potentially skew the image normalization for inference. To prevent these problems, we trained and incorporated DL modules within Deeplasia to automatically extract the hand from the scan by masking the background. Some examples of the results of our preprocessing on dysplastic hands are shown in Fig. 3. The details of our hand segmentation approach are described in Supplementary Material 1. The training masks and the code for the hand segmentation are publicly available via Rassmann et al. [31] and github.com/aimi-bonn/ hand-segmentation, respectively.

#### Bone age model training

As a baseline approach for the BA model, we followed the design principle winning the 2017 RSNA Pediatric BA ML Challenge [15, 16]. The model architecture, outlined in Supplementary Material 2, is composed of a fully convolutional neural network (CNN) as a feature extractor, channel-wise average pooling of the extracted features, and concatenation of a representation of the patient’s sex inflated to 32 neurons (we further discuss the effect of sex on BA assessment in Supplementary Material 2). The results are passed through a variable set of fully-connected (FC) layers to achieve a final prediction. We employ *EfficientNets* [32] as backbone feature extractors. In comparison to previously proposed end-to-end learning methods [16, 33], our applied average pooling reduces the dimensionality of the learned features and, thus, decreases the model size. For example, the largest of our BA models has a feature dimensionality of 1,792 resulting in a total network size of 23 × 10^6^ parameters, while the configuration proposed by Torres et al. [33] uses a feature dimensionality of 33,712 and 82 × 10^6^ parameters. All the details of our training procedure for reproducing the models are described in Supplementary Materials 2 and 3, and the open-source code for training the BA models is available at github.com/aimi-bonn/Deeplasia and deeplasia.de.

#### Model explorations for building an ensemble

**Fig. 5:**
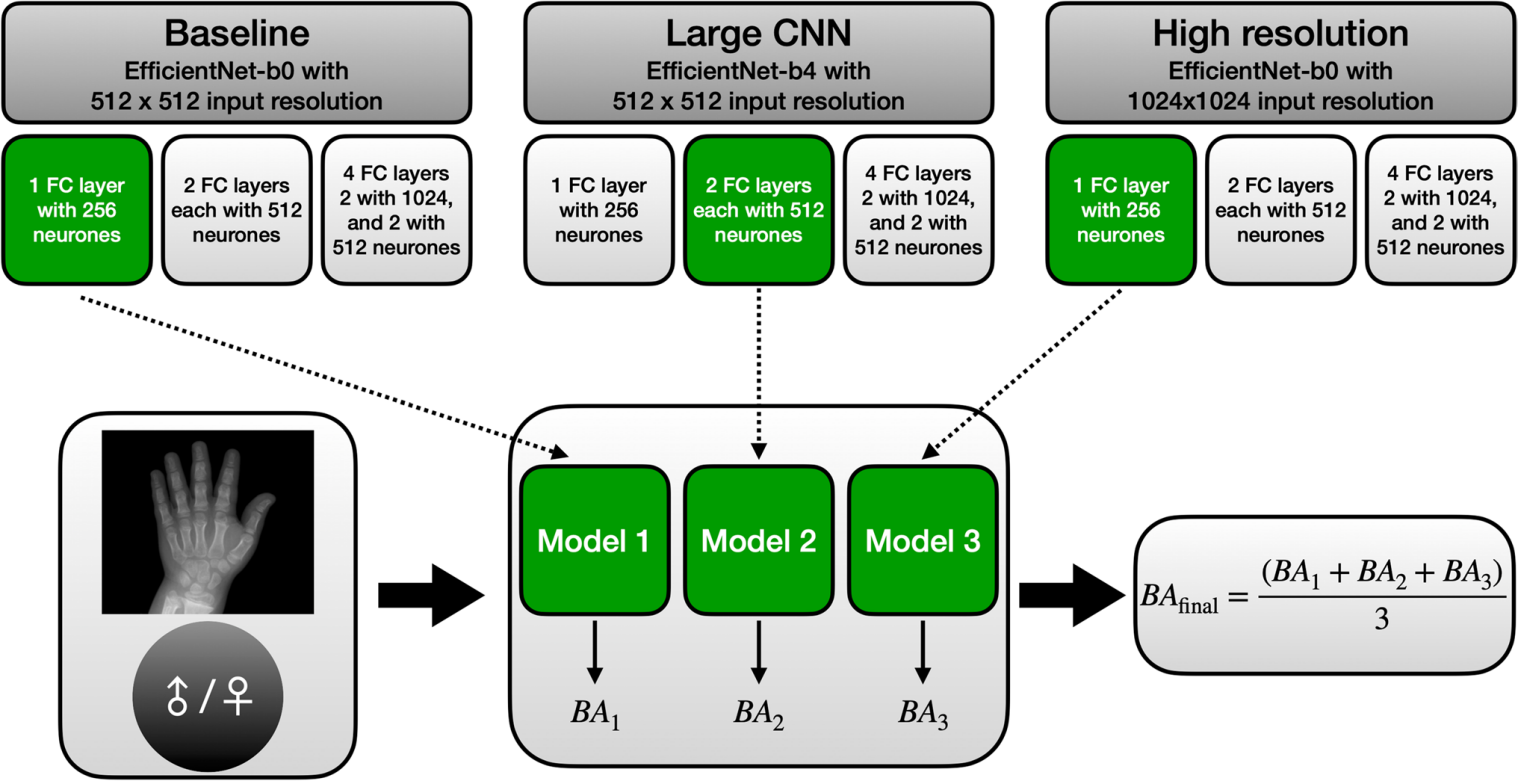
The flowchart describes the procedure for experimenting with different training configurations when building a model ensemble. We experimented with three different training conditions (*baseline*, *large CNN*, and *high-resolution*) each with three sets of FC-layer combinations. Out of these nine CNN models, we selected three (one per training condition, shown in green in the figure) based on their performance on the validation data set. See the “Design and development of Deeplasia” section and Supplementary Material 3 for further details. *BA* bone age, *CNN* convolutional neural network, *FC* fully connected

To build the model ensemble for Deeplasia, we experimented with three different training conditions: (a) *baseline*: *EfficientNet-b0* with 512 × 512 input resolution, (b) *large CNN*: *EfficientNet-b4* with 512 × 512 input resolution, and (c) *high-resolution*: *EfficientNet-b0* with 1024 × 1024 input. For each of these conditions, we trained models with three sets of FC layers: [256], [512, 512], and [1024, 1024, 512, 512]. Therefore, in total, we trained nine CNN models for BA estimation. A flowchart describing this procedure is shown in Fig. 5 and the details of our training experimentations are described in Supplementary Material 3.

To choose the models for building an ensemble, we analyzed the pairwise correlations between the predicted BA of these nine models (see Supplementary Material 3). This revealed that there is a higher correlation between the predictions of the models within each training condition (i.e. the *baseline*, *large CNN*, and *high-resolution*) compared to the ones between the models across these conditions. As dissimilar prediction patterns in a model ensemble are advantageous due to partial compensation of predictive errors, it is beneficial to construct an ensemble composed of models across different training conditions. Consequently, we chose to pick the best-performing model from each of the three training conditions (green boxes in Fig. 5) for building our model ensemble. The final BA is the average of the results from these three models.

### Evaluation methods

#### Metrics and statistical analysis

For model selection and benchmarking, the mean absolute difference (MAD) was used. It is calculated as the *L*_1_-norm of the difference between predicted BA $\widehat{Y}={\left({\widehat{y}}_{1},{\widehat{y}}_{2},\dots ,{\widehat{y}}_{n}\right)}^{T}$ and the respective ground truth $Y={\left({y}_{1},{y}_{2},\dots ,{y}_{n}\right)}^{T}$:

$$\mathrm{MAD}\left(\widehat{Y},Y\right)=\frac{1}{n}{\Vert \widehat{Y}-Y\Vert }_{1}=\frac{1}{n}\sum_{i=1}^{n}\left|{\widehat{y}}_{i}-{y}_{i}\right|$$

Further, the root-mean-square error (RMSE) was used as a metric that is more sensitive to outliers. It is defined as:

$$\mathrm{RMSE}\left(\widehat{Y},Y\right)=\sqrt{\frac{1}{n}{\Vert \widehat{Y}-Y\Vert }_{2}^{2}}=\sqrt{\frac{1}{n}\sum_{i=1}^{n}{\left({\widehat{y}}_{i}-{y}_{i}\right)}^{2}}$$

For the statistical analysis, we assume the error to be normally distributed and, thus, derive the confidence intervals of the RMSE from the corresponding *χ*^2^ distribution. As an additional, clinically more interpretable metric, we define a 1-year accuracy. Let **1**_condition_ denote the indicator function (a function that evaluates to 1 if and only if *condition* is true) and assume the BAs *Y*^ˆ^ and *Y* to be denoted in years, then

$${\mathrm{Accuracy}}_{1-\mathrm{year}}\left(\widehat{Y},Y\right)=\frac{1}{n}\sum_{i=1}^{n}{1}_{\left|{\widehat{y}}_{i}-{y}_{i}\right|\le 1}$$

Note that we do not conduct a symbolic perturbation, so the measure is conservative with regard to the model performance as the models, in contrast to human raters, are unlikely to assign integer BAs.

#### Longitudinal analysis

To detect small changes in development (slowdowns or growth spurts), BA measurements are required to have high test–retest reliability. Directly measuring the test–retest reliability would require a dedicated imaging session which would be unethical due to the unnecessary radiation exposure. However, assuming linear progress of the BA over time, the test–retest reliability can be estimated retrospectively from regular check-ups within the testing cohort. For estimating the upper bound of the expected error in assessing the BA, the method proposed by Thodberg and Sävendahl [34] was used. No patients were excluded due to therapies or other interventions. The potential variability in growth patterns due to the disorders of the patients included in the analysis might give a very non-linear growth pattern. To account for this, we set the maximum time difference for the derivation triplets to 14 months, the lowest threshold to achieve *n* ≥100 triplets. For analyzing the rater performance, only triplets derived from either the clinical ratings or from a single rater within the blinded re-rating session were included to avoid rater-rater biases or biases between clinical and blinded reviews.

#### Attention maps

To cast light on the decision-making process of our end-to-end method, we produce the so-called attention maps by calculating the absolute value of the gradient of the predicted BA with respect to the input image. These maps highlight the regions in the image that, according to the models, are important for assessing BA (see Supplementary Material 4 for further details on generating these maps).

## Results

### Performance on the test set of the Radiological Society of North America

On the RSNA test set comprising 200 radiographs, Deeplasia achieved a MAD of 3*.*87 months, RMSE of 5*.*14 months, and a 1-year accuracy of 98*.*5% (Table 1). Interestingly, even the three individual models of our ensemble (see Supplementary Material 3) achieved test accuracies (MAD of 4*.*2, 4*.*1, and 4*.*3 months for the best-performing models of the *baseline*, *large CNN*, and *high-resolution training conditions*, respectively) comparable to other approaches incorporating human priors.

**Table 1:** Deeplasia and inter-rater accuracies across different test datasets^a^

Dataset	Num. ref. ratings	*n*	Deeplasia (months)		Inter-rater (months)	
			MAD	RMSE [95% confidence interval]	MAD	RMSE [95% confidence interval]
RSNA	6	200	3.9	5.1 ([4.7, 5.7])	4.8–7.0^b^	-
DHA	2	1,383	5.8	7.7 ([7.4, 8.0])	4.4	7.0 ([6.8, 7.3])
GDBD	2	702	6.0	7.7 ([7.3, 8.1])	9.5	12.8 ([12.2, 13.6])
GDBD (gen. dis.)	2	568	5.8	7.5 ([7.1, 7.9])	9.5	12.8 ([12.1, 13.6])

*DHA* Los Angeles Digital Hand Atlas, *GDBD* German Dysplastic Bone Dataset, *GDBD (gen. dis.)* the subset of the GDBD with molecularly confirmed genetic diagnosis of one of the seven skeletal dysplasias in this study, *MAD* mean absolute difference, *RMSE* root mean squared error, *RSNA* Radiological Society of North America

^a^Lower MAD and RMSE indicate higher accuracy, ^b^Estimated range for the accuracies of the assessed single raters

### Performance on the Digital Hand Atlas dataset

To assess the generalizability to external test cohorts and potentially unseen ethnicities, we evaluated Deeplasia on the Digital Hand Atlas dataset [28, 29]. We used 1,383 radiographs from children (age 0–18 years) with different ethnic backgrounds and their corresponding BA ratings. On this dataset, Deeplasia achieved a MAD of 5.81 months, RMSE of 7.67 months, and a 1-year accuracy of 92*.*9% (see the second row of Table 1). Note that for this dataset, the ground truth BA estimates are based on two rather than six raters for the RSNA test set.

### Performance on the German Dysplastic Bone Dataset

Finally, we evaluated the performance of Deeplasia on the German Dysplastic Bone Dataset to assess the generalization of Deeplasia to patients with skeletal dysplasias. Overall, this dataset contains 568 images from patients with a molecularly confirmed genetic disorder, 55 images from patients with IUGR, and 79 images from individuals without any genetically diagnosed dysplasia, but who had been referred to pediatric endocrinologists due to a suspected growth anomaly. All reference BA ratings were performed by the same two raters (K.M. and A.K.).

**Fig. 6:**
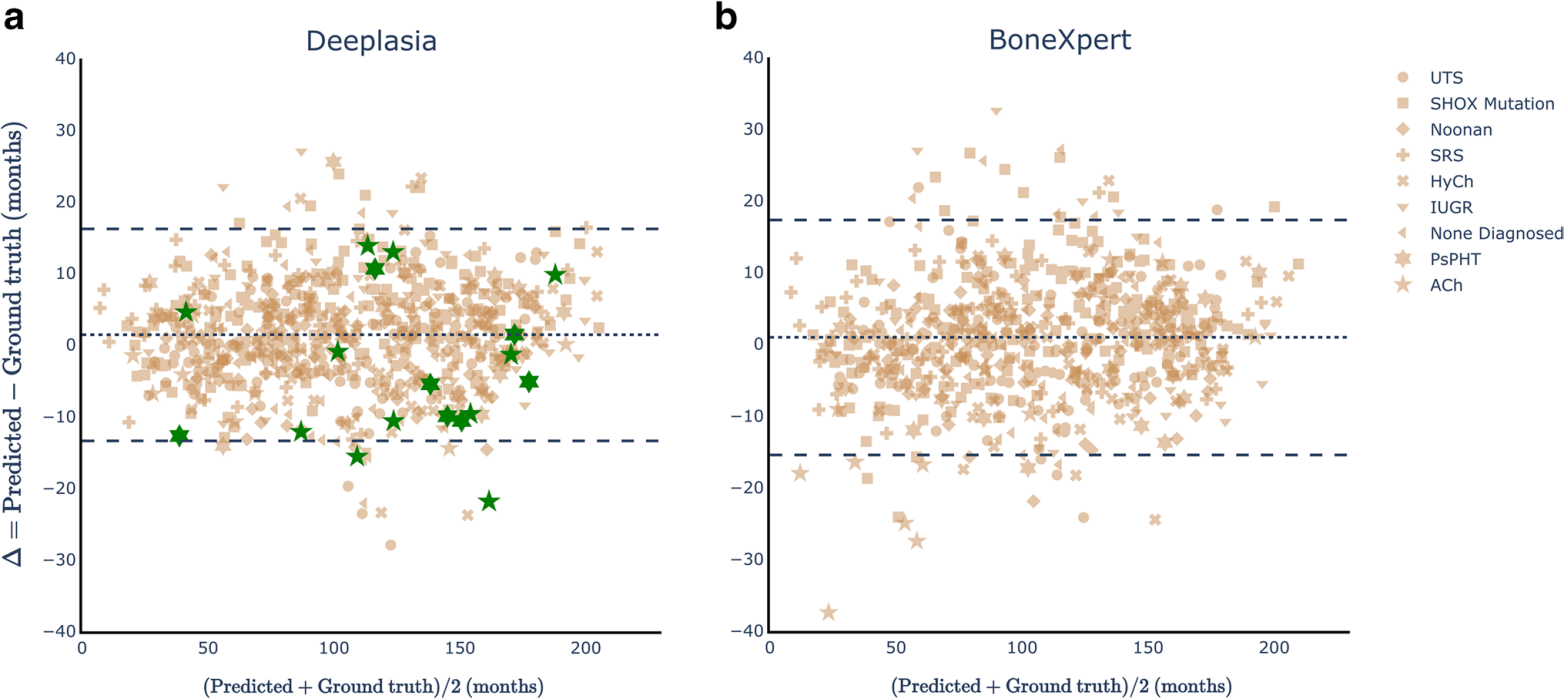
Bland–Altman plots showing the performances of Deeplasia (**a**) and BoneXpert (**b**) on the German Dysplastic Bone Dataset (GDBD) hand radiographs. **a** The MAD of Deeplasia (for all the 702 GDBD radiographs) is 6.0 months. The cases rejected by BoneXpert (11 achondroplasia and seven pseudohypoparathyroidism) are indicated (*green*). For these cases, the performance of Deeplasia is similar to that for other cases and 16 out of 18 of them are within the 95% confidence interval (*broken* *lines*). **b** The MAD of BoneXpert (for 684 radiographs) is 6.3 months. Note that even for the achondroplasia cases that were not rejected by BoneXpert, a drop in its performance is visible for children below 5 years old. *ACh* achondroplasia, *HyCh* hypochondroplasia, *IUGR* intrauterine growth restriction, *MAD* mean absolute difference, *PsHPT* pseudohypoparathyroidism, *SHOX mut*. mutation of the *SHOX* gene, *SRS* Silver-Russel syndrome, *UTS* Ullrich-Turner syndrome. An interactive version of the Bland–Altman plot for Deeplasia can be accessed via https://aimi-bonn.github.io/website/deeplasia/results.html and deeplasia.de

Comparing the predictions of Deeplasia and the ground truth estimates defined by the average of two raters, the model ensemble achieved a MAD of 5*.*96 months, RMSE of 7*.*67 months, and a 1-year accuracy of 90*.*2% for the full set and 5*.*84 months (MAD), 7*.*48 months (RMSE), and 90*.*1% (1-year accuracy) for the subset of patients with molecularly confirmed disorders. These values (also listed in the third and fourth rows of Table 1) are similar to those from the performance on the Digital Hand Atlas dataset and in the range of the single rater estimated in the annotation of the RSNA BA challenge [15]. Consequently, the error of the model ensemble with respect to the average of two reference ratings is smaller than the assessed inter-rater error (Table 1). In Fig. 6, we illustrate the Bland–Altman plot for Deeplasia. It shows the difference between the BA predictions from Deeplasia and the reference values (from the two raters) vs. the average of the two methods. The mean difference of the two methods is ∆ =  + 1*.*4 months (shown by a broken line), and the plot reveals no systematic over- or underestimation of the BAs for different skeletal disorders. The difference between the predicted BA and the reference ratings is within 1.96 standard deviations (i.e. the 95% confidence interval) for 95*.*6% of the predicted BAs.

Analyzing the models’ predictive error for individual disorders, listed in Table 2, shows no significant drop in performance in comparison to the children with no diagnosed disorder. However, a tendency of increased RMSE and MAD is observed for achondroplasia, hypochondroplasia, and pseudohypoparathyroidism, while a significantly decreased error is observed for Noonan and Ullrich-Turner syndrome. This may be attributed to the accuracy of the reference grading, given that the inter-rater errors (columns 7 and 8 of Table 2) are also higher for achondroplasia, hypochondroplasia, and pseudohypoparathyroidism and lower for Noonan syndrome and Ullrich-Turner syndrome. Of note, for each disorder, the average error of the model in comparison to a single manual rating (columns 5 and 6 of Table 2) is smaller than the average difference between the two manual raters. Hence, our model ensemble is at least as accurate as the assessed human raters for all assessed disorders and, at the same time, retains accuracy for severe skeletal dysplasias (achondroplasia and pseudohypoparathyroidism), while inter-rater disagreement is increased for these conditions.

**Table 2:** The accuracy of Deeplasia on the German Dysplastic Bone Dataset

Disorder	*n*	Deeplasia w.r.t. two raters (months)		Deeplasia w.r.t. a single rater (months)		Inter-rater error (months)	
		MAD	RMSE	MAD	RMSE	MAD	RMSE
ACh	25	7.3	9.2 ([7.2, 12.7])	10.0	13.1 ([10.9, 16.2])	13.3	18.5 ([14.5, 25.5])
HyCh	44	7.2	9.5 ([7.9, 12.0])	8.4	11.8 ([10.3, 13.9])	11.6	13.9 ([11.5, 17.6])
Noonan	80	4.3	5.6 ([4.8, 6.6])	7.5	8.0 ([7.2, 9.0])	8.9	11.5 ([9.9, 13.6])
PsHPT	30	7.5	8.8 ([7.1, 11.8])	10.3	13.9 ([11.8, 17.0])	13.9	21.5 ([17.2, 28.7])
SHOX mutation	198	5.9	7.5 ([6.8, 8.3])	7.5	9.6 ([9.0, 10.3])	9.5	12.0 ([11.0, 13.4])
Silver-Russell	69	6.2	7.7 ([6.6, 9.2])	7.7	9.6 ([8.6, 10.8])	7.7	11.5 ([9.8, 13.8])
Ullrich-Turner	122	5.2	6.9 ([6.1, 7.9])	6.4	8.7 ([8.0, 9.6])	8.4	11.5 ([9.6, 12.3])
IUGR	55	7.2	8.9 ([7.5, 11.0])	8.8	10.8 ([9.6, 12.5])	8.5	12.2 ([10.3, 15.0])
None diagnosed	79	6.3	8.1 ([7.0, 9.6])	9.3	10.5 ([9.5, 11.8])	10.0	13.5 ([11.6, 15.9])

Accuracy is shown with respect to the average bone age rating of two raters (columns 3 and 4) and a single rater (columns 5 and 6), plus the inter-rater errors (columns 7 and 8), lower MAD and RMSE errors mean higher accuracy. *n* refers to the number of individual radiographs per disorder. The RMSE is stated with the 95% confidence interval. *ACh* achondroplasia, *GDBD* German Dysplastic Bone Dataset, *HyCh* hypochondroplasia, *IUGR* intrauterine growth restriction, *MAD* mean absolute difference, *PsHPT* pseudohypoparathyroidism, RMSE root mean squared error, *w.r.t* with respect to

For a quantitative comparison between a bone segmentation-based method and our end-to-end approach, we applied the commonly-used BoneXpert software [25] on the hand radiographs contained in the German Dysplastic Bone Dataset (Fig. 6). BoneXpert failed to assess the BA of 18 radiographs in this dataset (11 achondroplasia and seven pseudohypoparathyroidism). For the remaining 684 radiographs of the German Dysplastic Bone Dataset, BoneXpert achieved a MAD and an RMSE of 6.3 and 8.4 months, respectively, while (for this subset) Deeplasia achieved a MAD of 5.9 months and an RMSE of 7.6 months. The performance of BoneXpert for the individual disorders in the German Dysplastic Bone Dataset is given in Supplementary Material 5. On the other hand, for the 18 cases rejected by BoneXpert, the MAD of Deeplasia is 9.4 months and its RMSE is 10.8 months.

### Performance on longitudinal data

In clinical scenarios, determining BA is not only important for receiving an initial diagnosis but also for monitoring development and maturation. This requires a high test–retest reliability for the measured BA. We retrospectively estimated the test–retest reliability from regular check-ups within our cohort, employing the method proposed by Thodberg and Sävendahl [34]. In brief, this method assumes a linear progress of BA between two measurements and compares the measured BA to the interpolation between adjacent BA estimates.

**Fig. 7:**
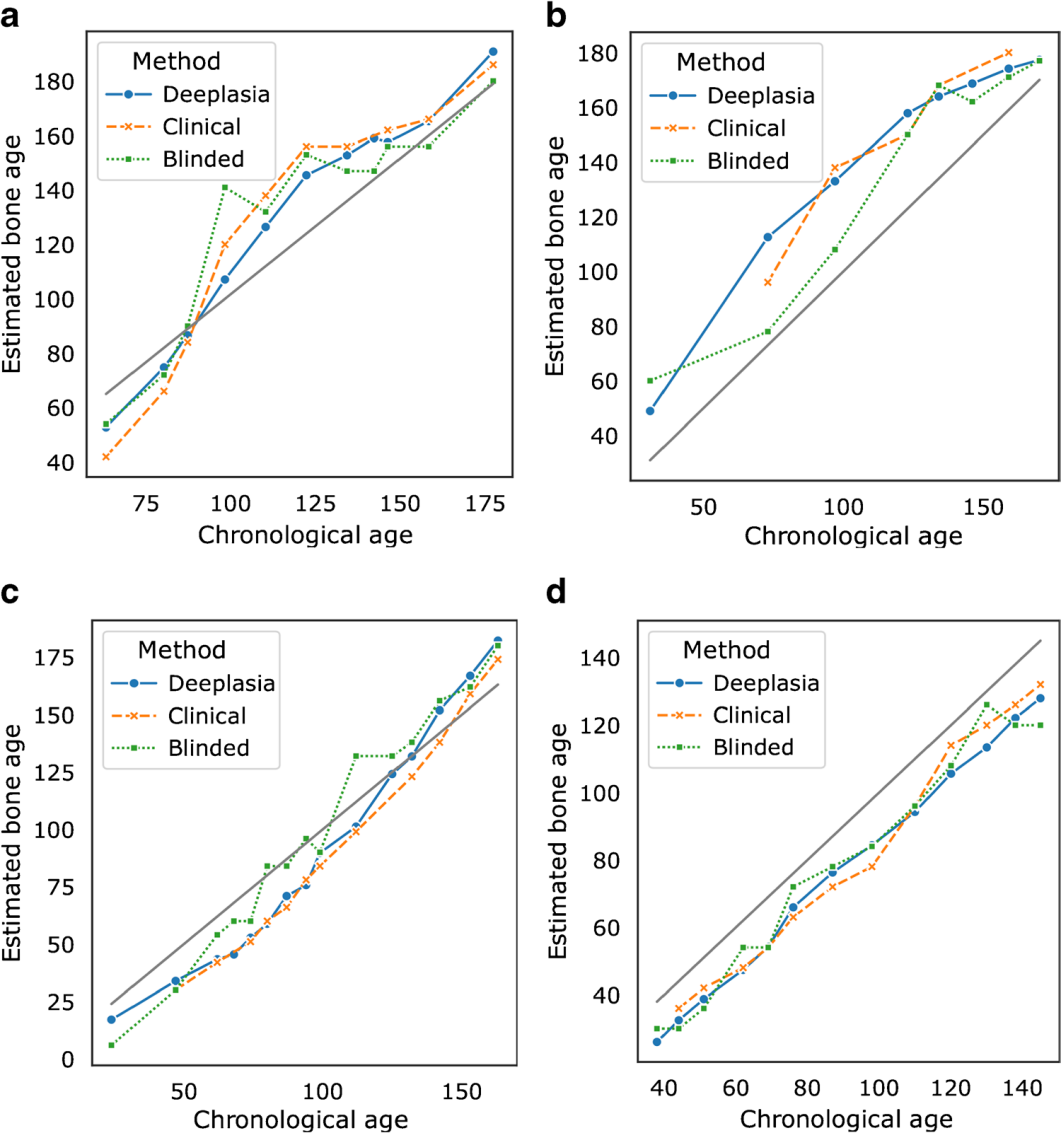
Plots of bone age maturation progress of individual patients within the German Dysplastic Bone Dataset estimated by Deeplasia, the clinical, and a blinded manual assessment. Bone age and chronological age are denoted in months. **a**, **b** Boys with hypochondroplasia (**a**) and pseudohypoparathyroidism (**b**). **c**, **d** Girls with Noonan (**c**) and Ulrich-Turner (**d**) syndromes

The results from this analysis are summarized in Table 3 and four examples are shown in Fig. 7. Based on the German Dysplastic Bone Dataset, we estimated the test–retest error on patients with genetic disorders to be at most 2*.*74 months (95% confidence interval [2*.*46*,* 3*.*09], *n* = 149). Comparing our results to the ground truth rating shows that the precision of Deeplasia is on par with clinical assessment. Nevertheless, in the clinical scenario, the patient’s identity, diagnosis, and BA results from previous examinations are known and can be used to smooth the next reported BA. If the ratings are conducted blinded and in a randomized order without additional information, the precision of the human BA reading drops significantly (Table 3) and the noise in manual BA assessment is clearly visible (Fig. 7). Thus, automatic BA prediction using Deeplasia is significantly more precise and reliable than a manual rating in a blinded scenario.

**Table 3:** The test–retest precision of Deeplasia

Rating method	Precision (months)	
	Full dataset (*n*=149)	With clinical BA (*n*=106)
Deeplasia	2.7 (2.5, 3.1)	2.4 (2.1, 2.8)
Clinical rating	-	2.6 (2.3, 3.0)
Blinded rating	5.6 (4.9, 6.2)	5.8 (5.1, 6.7)

The clinical and a blinded manual bone age rating is estimated on patients with genetically-confirmed disorders (i.e. excluding intrauterine growth retardation and  non-diagnosed cases) within the German Dysplastic Bone Databse (GDBD). *n* refers to the number of images within the GDBD for which the interpolation residuals could be estimated. The precision is stated with a 95% confidence interval. *GDBD* German Dysplastic Bone Dataset

### Deeplasia’s attention maps

**Fig. 8:**
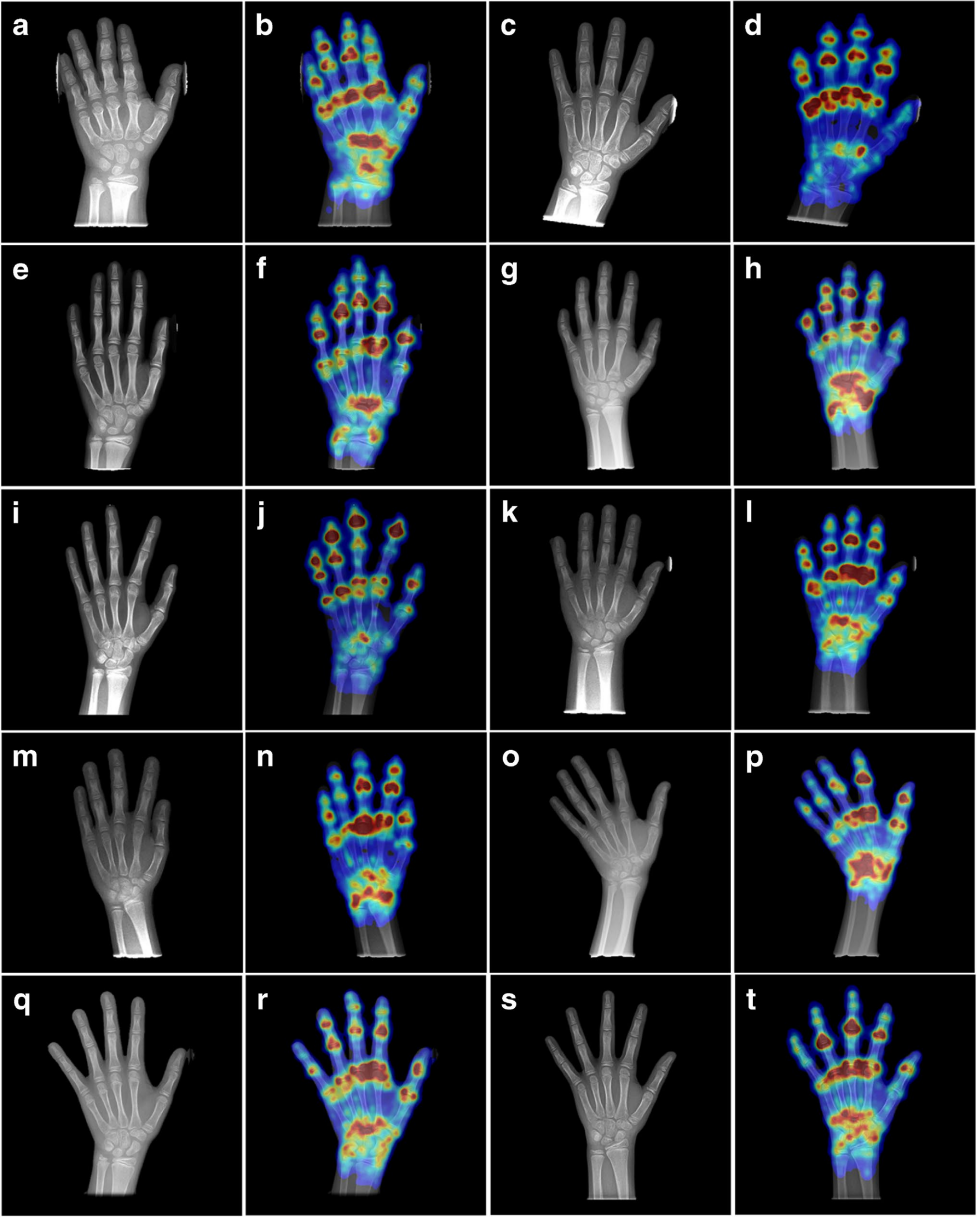
Example attention maps from Deeplasia. **a**, **b** A 7-year-old girl with achondroplasia. **c**, **d** A 10-year-old girl with hypochondroplasia. **e**, **f** A 10-year-old girl with intrauterine growth restriction. **g**, **h** A 10-year-old girl with Noonan syndrome. **i**, **j** An 11-year-old girl with pseudohypoparathyroidism. **k**, **l** A 10-year-old girl with a mutation of the *SHOX* gene. **m**, **n** A 10-year-old girl with Silver-Russell syndrome. **o**, **p** A 9-year-old girl with Ullrich-Turner syndrome. **q**, **r** A 9-year-old girl with no genetic disorder. **s**, **t** A 9-year-old girl with no genetic disorder. For each example, the radiograph (**a**, **c**, **e**, **g**, **i**, **k**, **m**, **o**, **q**, **s**) and the attention map (**b**, **d**, **f**, **h**, **j**, **l**, **n**, **p**, **r**, **t**) is shown. These maps show that the attention of the models is mainly on the phalangeal and metacarpal joints, as well as the carpal bones, i.e. the regions relevant for bone age assessment. Larger versions of these images, as well as the exact bone age measurements, are available in Supplementary Material 4

In Fig. 8, we illustrate ten examples of the resulting attention maps from Deeplasia. These maps show that the attention of the models is mainly on the phalangeal and metacarpal joints, as well as the carpal bones, i.e. the regions relevant for BA assessment.

## Discussion

Deeplasia achieved a competitive MAD of 3.87 months on the RSNA test set, which is on par with the current state-of-the-art (3.91 months, [18]) and tools cleared for clinical use (4*.*1 months, [20, 25]). This demonstrates that our prior-free learning approach is as powerful as other approaches that require additional annotations, ROI extractions, or human priors.

On the German Dysplastic Bone Dataset—a new dataset comprising radiographs with skeletal dysplasias—Deeplasia achieved a MAD of 5.96 months, RMSE of 7*.*67 months, and a 1-year accuracy of 90*.*2% (based on two reference ratings). These results are slightly better than those reported by Wang et al. [27] in their study of a cohort consisting of 745 Chinese patients. They report a MAD of 6.96 months, RMSE of 9.12 months, and a 1-year accuracy of 84.6%. However, their cohort included a wider range of developmental growth disorders (including 20 different classes).

When assessing the performance of the commonly-used BoneXpert software [25] on the hand radiographs contained in the German Dysplastic Bone Dataset, we found that BoneXpert rejected 11 out of 25 (44%) achondroplasia cases and 7 out of 30 (23%) pseudohypoparathyroidism cases. The BoneXpert rejection rate for achondroplasia is in agreement with the expected ≈50% (personal communication with H. H. Thodberg, March 2023). While for the 18 cases rejected by BoneXpert, there is a drop in the overall performance of Deeplasia (MAD=9.4 and RMSE=10.8 months); its error is still significantly smaller than the inter-rater error (Table 2). Also, as is visible in the Bland–Altman plot, Deeplasia’s predictions for these 18 cases show no significant deviation from the ground truth. In fact, 16 out of 18 of these cases lie within the 95% (or 1*.*96*σ*) confidence intervals, and the other two cases are only 2*.*1*σ* and 2*.*9*σ* from the ground truth. We remind the reader that the ground truth values are the average of two experts with a total of 60 years of experience in pediatric BA assessment. However, it would be necessary to further study the performance of Deeplasia on larger cohorts, especially to test on a larger number of achondroplasia and pseudohypoparathyroidism cases.

A general concern regarding medical AI is to understand its decision-making process [35]. While methods relying on the segmentation of individual bones offer a higher degree of explainability compared to end-to-end learning methods, this study shows that the latter is successful in analyzing dysmorphic bones for which the former methods do not always work. However, the generalization process of the AI from normal to abnormal bones might appear difficult to comprehend. We have shed light on the decision-making process of our end-to-end method by producing the so-called attention maps, illustrated in Fig. 8. These maps reveal that the models primarily focus their attention on the phalangeal and metacarpal joints, along with the carpal bones, which are the pertinent areas for assessing bone age. In addition, the observable patterns in the attention maps of the dysplastic hands remain unaltered in comparison to the hands with no genetic disorder. This shows that the activation patterns within the model are invariant to the dysmorphologies represented in the German Dysplastic Bone Dataset and the extracted features remain unaffected by the anomalies. Combined with the results of the unaltered performance, this shows the generalizability of Deeplasia to the presence of skeletal disorders in the input images.

While there have been some studies employing DL-based techniques on medical images of patients with rare genetic diseases (e.g., [36–38].), this field is still understudied, perhaps mainly due to the inherently small quantity of data available for such disorders. The current study is limited to only seven different genetic bone diseases. Hence, future work should expand the current dataset to a broader set of disorders and patients with varying ethnic backgrounds (For e.g., via support from FAIR [39] sources such as the GestaltMatcher Database [40]).

## Conclusion

As patients with skeletal dysplasias are an important group requiring bone age assessment, it is vital to ensure the applicability and generalizability of automated approaches to these patients in dedicated studies such as this work. We have demonstrated that our prior-free deep-learning ensemble system, Deeplasia

- achieves a competitive performance on the RSNA BA test dataset composed of predominantly healthy patients,
- generalizes to patients from unseen cohorts and with a variety of genetically-confirmed skeletal dysplasias, and
- is applicable to longitudinal data from patients with skeletal dysplasias for progressive growth monitoring.

We have provided the codes we developed for our model ensemble to the community for scrutiny and reuse in their research.

### Supplementary Information

Below is the link to the electronic supplementary material.

Supplementary file1 (PDF 2898 KB)

## Data Availability

The RSNA datasets and the Los Angeles Digital Hand Atlas are publicly available.

## Code availability

All the codes developed for training the models in this study as well as the ones used for preprocessing of the data are open-source and are publicly available at github.com/aimi-bonn/

Deeplasia and github.com/aimi-bonn/hand-segmentation, respectively, and via Deeplasia.de.
